# Discovery of potent STAT3 inhibitors using structure-based virtual screening, molecular dynamic simulation, and biological evaluation

**DOI:** 10.3389/fonc.2023.1287797

**Published:** 2023-11-02

**Authors:** Weifeng Liu, Zhijie Chu, Cheng Yang, Tianbao Yang, Yanhui Yang, Haigang Wu, Junjun Sun

**Affiliations:** ^1^ Department of Hepatobiliary and Pancreatic Surgery, The First Affiliated Hospital, and College of Clinical Medicine of Henan University of Science and Technology, Luoyang, Henan, China; ^2^ Department of Emergency Trauma Surgery, First Affiliated Hospital, College of Clinical Medicine, Henan University of Science and Technology, Luoyang, Henan, China; ^3^ School of Life Sciences, Henan University, Kaifeng, Henan, China

**Keywords:** STAT3, structure-based virtual screening, molecular docking, molecular dynamic simulation, gastric cancer

## Abstract

**Introduction:**

Signal transducer and activator of transcription 3 (STAT3) is ubiquitously hyper-activated in numerous cancers, rendering it an appealing target for therapeutic intervention.

**Methods and results:**

In this study, using structure-based virtual screening complemented by molecular dynamics simulations, we identified ten potential STAT3 inhibitors. The simulations pinpointed compounds 8, 9, and 10 as forming distinct hydrogen bonds with the SH2 domain of STAT3. *In vitro* cytotoxicity assays highlighted compound 4 as a potent inhibitor of gastric cancer cell proliferation across MGC803, KATO III, and NCI-N87 cell lines. Further cellular assays substantiated the ability of compound 4 to attenuate IL-6-mediated STAT3 phosphorylation at Tyr475. Additionally, oxygen consumption rate assays corroborated compound 4's deleterious effects on mitochondrial function.

**Discussion:**

Collectively, our findings position compound 4 as a promising lead candidate warranting further exploration in the development of anti-gastric cancer therapeutics.

## Introduction

Gastric cancer remains a significant global health challenge, with over one million newly diagnosed cases in 2020 and an estimated 769,000 deaths worldwide (1). While gastric cancer can be classified into three histological subtypes: intestinal, diffuse, and mixed, a recent genomics-based analysis from the TCGA database delineated four molecular subtypes: Epstein-Barr virus-positive (EBV), microsatellite instability (MSI), chromosomal instability (CIN), and genomically stable (GS) (1, 2). Currently, there is no universally accepted gold standard for gastric cancer treatment. Depending on the disease stage and progression, therapeutic approaches range from surgical interventions and cytotoxic therapies to targeted treatments (3, 4). In the realm of targeted therapies, various strategies, including tyrosine kinase inhibitors, cellular structure remodeling, DNA damage repair protein targeting, and immunotherapy, have been explored to enhance gastric cancer patient survival (5). Despite recent advancements in oncological interventions, existing data underscore the need for further research to refine and optimize the therapeutic options for gastric cancer.

Elevated activity of signal transducer and activator of transcription 3 (STAT3) is evident in numerous cancers and plays a pivotal role in inflammation-associated tumorigenesis (6, 7). In gastric cancer, STAT3 intricately governs cell proliferation, differentiation, apoptosis, and angiogenesis (8–10), making it an attractive target for drug development. STAT3 is constitutively activated, primarily involving two conserved amino acid residues, Tyr-705 and Ser-727 (11). Phosphorylation of these residues underpins the sustained hyperactivity of STAT3. Common upstream effectors that facilitate STAT3 phosphorylation include Janus-activated kinase (JAK), Src kinase, and epidermal growth factor receptor (EGFR), which promote STAT3 dimerization via the Src homology 2 (SH2) domain. These STAT3 dimers then translocate to the nucleus, orchestrating the expression of downstream genes, such as MYC, BCL2, IL10, MCL1, IL11, MMP9, MUC1, EGFR, COX2, IFNG, and VEGF. In contrast to Tyr-705 phosphorylation, Ser727 phosphorylation directs signaling via non-canonical STAT3 activation pathways involving mitogen-activated protein kinase (MAPK), extracellular regulated protein kinases (ERK1/2), c-Jun, N-terminal kinase (JNK), c-kit (12), and p38 (13, 14). These effectors are widely recognized as pivotal regulators that alter mitochondrial oxidative phosphorylation, thereby affecting tumor proliferation. Targeting STAT3 with small molecules is a promising strategy for halting the progression of gastric cancer.

**Figure 1:**
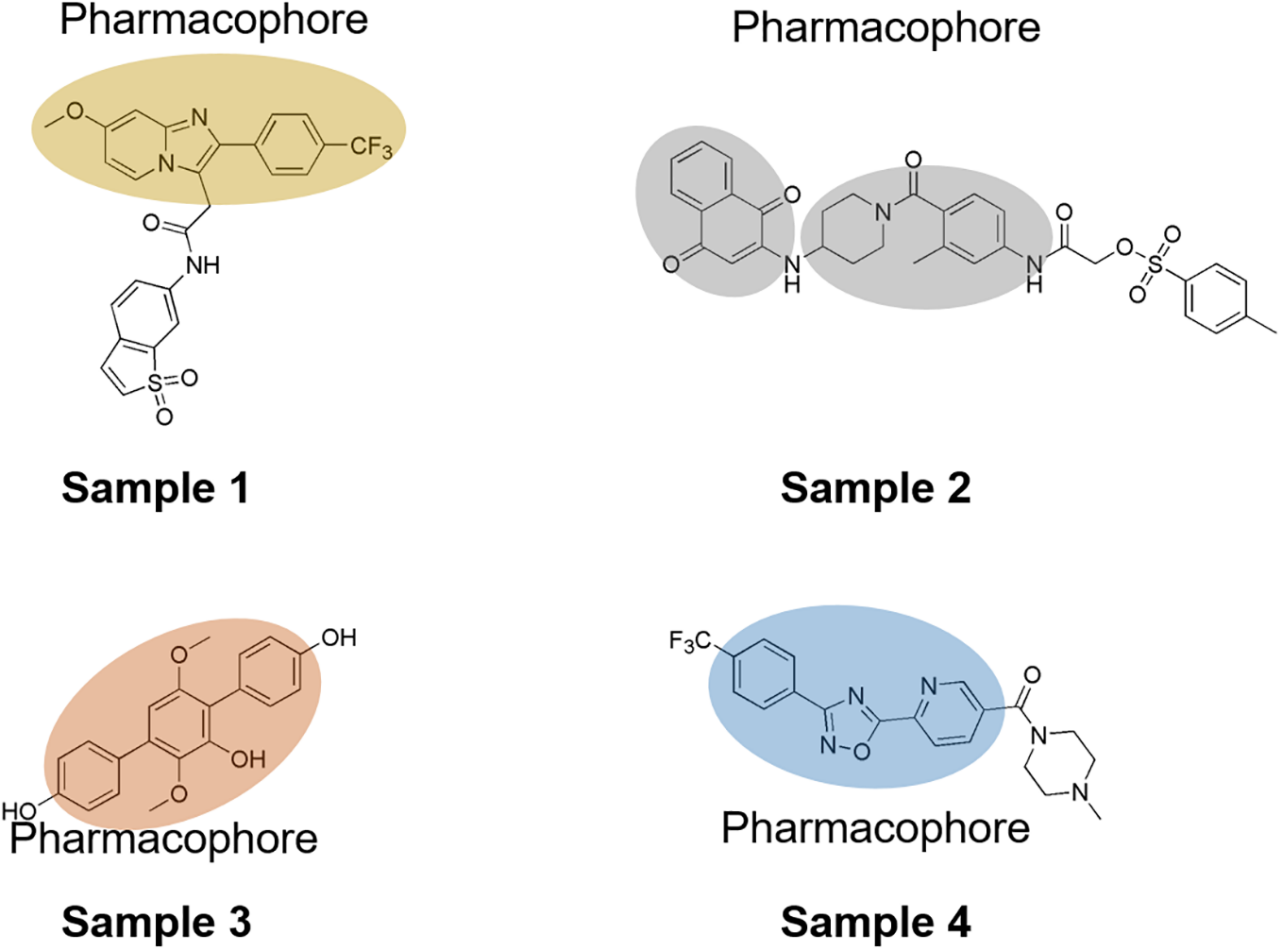
Current STAT3 inhibitors for gastric cancer therapy.

Over the past decade, numerous STAT3 inhibitors have been developed, including peptides, peptidomimetics, and oligonucleotides (**Figure 1**). However, many of these inhibitors face challenges in clinical application because of their low affinity, suboptimal cell permeability, and poor bioavailability. To address these limitations, innovative strategies targeting the N-terminal domain, Cys and p-Tyr, SH2 domain, and DNA-binding domain have emerged. Notably, the SH2 domain showed a higher binding affinity for non-peptide molecules, such as S3I-201, Stattic, and STA-21 (15). These inhibitors effectively targeted subpockets encompassing the phosphorylated Tyr705-binding pocket, the Leu706 subsite, and a hydrophobic side pocket. Chen et al. reported that ciclopirox could suppress the phosphorylation of STAT3 at Tyr705 and 727 for gastric cancer therapy (16); Boengler, Kerstin et al. disclosed that benzo[b]thiophene 1,1-dioxide (BTP) variants could attenuate the Tyr705 phosphorylation levels of STAT3 while enhancing the generation of reactive oxygen species (ROS) (17). Additionally, the sulfonamide derivative BP-1-102 exhibitspotent STAT3 binding affinity, inhibiting its DNA-binding activity (18, 19). Meanwhile, the quinone analog BPMP-28 is also known to irreversibly bind to STAT3, curtailing the proliferation of MDA-MB-231 and MDA-MB-468 at concentrations as low as 3 μM (20). Nonetheless, many of these inhibitors still grapple with issues of low solubility, limited efficacy, and adverse side effects. Thus, this study aimed to identify novel STAT3 inhibitors that may broaden the therapeutic arsenal for gastric cancer treatment.

In this study, we conducted a structure-based virtual screening using commercial small molecule databases, in which 10 small molecules with potential STAT3 inhibitory activity were identified. Subsequent biological assays demonstrated that four of these compounds effectively suppressed the proliferation of gastric cancer cells, including MGC803, NCI-N87, and KATO III cell lines. Luciferase activity assays revealed that these molecules successfully inhibited STAT3 binding to the SH2 domain, and a cellular thermal shift assay validated the binding efficiency of small molecules with STAT3. We also determined the biological effects of these STAT3 inhibitors on ATP production and oxygen consumption rate in gastric cancer cells. Collectively, our findings provide new insights into the drug development of STAT3 inhibitors and suggest that these small molecules could be potential lead compounds and chemical core structures for further chemical modifications to obtain potent STAT3 inhibitors.

## Experimental sections

### Cell culture

Various types of gastric cancer cell lines, including MGC803, NCI-N87, and KATO III, were obtained from the American Type Culture Collection (Manassas, VA, USA). The cells were cultured in Dulbecco’s Modified Eagle Medium (DMEM) containing 10% fetal bovine serum (FBS) and 100 U/mL penicillin-streptomycin. Cells were cultured in the humidified incubator with 5% CO_2_ at 37°C.

### Protein active pockets of STAT3 and molecular library

The crystal structure of STAT3 complexed with a small-molecule inhibitor (PDB ID: 6NJS) was obtained from the Research Collaboratory for Structural Bioinformatics (RCSB) protein database (https://www.rcsb.org/). The resolution of STAT3 is 2.70 Å. The STAT3 unit contains two domains: a DNA-binding domain and an SH2 domain. Prior to molecular docking, all co-crystallized ligands and water molecules were removed. The active pocket of STAT3 was set as the ligand-binding region located inthe SH2 domain.

The library contained three sections: InterBioscreen, Life Chemical-Bioactive Screening Compound Library, and Life Chemical-Synthetic Compounds. InterBioscreen (https://www.ibscreen.com/database) comprises of approximately 550,000 compounds. Life Chemicals offers a Bioactive Screening Compound Library of approximately 9,900 drug-like small molecules as a perfect starting point for phenotypic (cell-based) and target-based high-throughput screening (HTS) drug discovery research projects. In response to the current demand for drug discovery, Life Chemicals created a proprietary collection of dedicated Screening Libraries of over 14,600 synthetic compounds similar to natural compounds using the following two approaches:

### ComboNet architecture

We executed deep learning virtual screening using the ComboNet framework, an extension of the ChemProp software (21, 22). Atomic features include attributes such as atomic number, degree, formal charge, chirality, bonded hydrogen count, hybridization, aromaticity, and atomic mass. The bond features included the bond type (single/double/triple/aromatic), conjugation, ring association, and stereochemistry. The model employs a succession of message-passing steps to refine the atom representations. In each iterative step, an atom’s feature set is revamped by accumulating incoming messages, concatenating them with existing atom features, and subsequently engaging a singular neural network layer with a nonlinear activation function. After a predetermined set of message-passing iterations, the refined atom representations were aggregated to formulate a unique molecular representation, denoted as zA. The vector representation dimensionality was standardized to |z| = 100. The open-source codebase of ChemProp software is accessible at https://github.com/chemprop/chemprop.

### Molecular docking assays

Docking simulations were divided into two sections: UCSF DOCK using the DOCK balstermaster pipeline (23) and Autodock Vina using binging energy methods (24). For the UCSF DOCK simulation, SPHGEN was employed to generate matching spheres in the binding pocket and regenerate 45 matching spheres by default. The score used to evaluate the binding efficiency was the GID score, which we selected as the top 10% for the next autoDock Vina screening. All binding files were prepared using AutodockTools1.5.7, and the binding parameters of the pocket were set to (13.498, 54.117, 0.1) with size (72, 74, 48). Finally, Ambers score using UCSF DOCK6.0 was employed to identify potential molecules for subsequent biological assays. The threshold value was set as less than -20.0.

### Molecular dynamic simulation

Molecular dynamics simulations were performed using GROMACS to assess the stability of the protein-ligand complex. The requisite Topol files were generated via the Acpype server (www.bio2byte.be) using the GAFF force field. A simple point-charge (SPC) water model was incorporated into a cubic box housing the STAT3-ligand complex. Subsequently, appropriate amounts of sodium and chloride ions were introduced to neutralize the charge. The system then underwent a 1000-step energy minimization using the steepest descent algorithm. An NVT ensemble was employed for 100 ps at 300 K, followed by a 100 ps NPT simulation using a Parrinello-Rahman barostat, ensuring a consistent pressure of 1 atm. Each complex underwent 50 ns molecular dynamics simulation, with the atomic coordinates of the protein-ligand system captured every 100 ps for subsequent analysis.

### ADMET properties of selected compounds

The ADMET properties of the selected 10 compounds were assessed using SwissADMET (http://www.swissadme.ch/index.php), inputting the compounds into the SMILES format (**SI Table S2**). The SMILES notations for these compounds were obtained using the OpenBabel software. All analysis parameters were maintained at their default settings.

### *In vitro* cytotoxicity assays

Gastric cancer cells were seeded in 96-well plates (501102, Nest) at a density of 10,000 cells/well and incubated overnight. Next, varying concentrations of small molecules (purchased from InterBioscreen) were administered to each well, and the plates were further incubated for 48 h. After this period, the medium was aspirated and the wells were treated with MTT solution (M8180, Solarbio, China), followed by a 30 min incubation. The supernatant was subsequently discarded, and 150 μL of dimethylsulfoxide (D8370, Solarbio, China) was added to solubilize the formazan crystals. The absorbance was measured at 570 nm using a microplate reader (Varioskan Flash, Thermo Scientific).

### STAT3 luciferase reporter assay

The STAT3 dual-luciferase reporter assay was conducted according to established protocols. HEK293T cells were transiently transfected with both luciferase reporter and Renilla plasmids using Lipofectamine 2000 (Invitrogen). After transfection, cells were seeded in 96-well plates at 10,000 cells/well and incubated overnight. IL-6 (20 ng/mL, P00022; Solarbio, China) served as a STAT3 activator. Luciferase assays were performed according to the guidelines of the Dual-Luciferase Reporter Assay System (RG021S; Beyotime). Luciferase readings were standardized against Renilla luciferase from a cotransfected control plasmid.

### ATP examination

Gastric cancer cells were seeded in 6-well plates at a suitable density and incubated overnight. Subsequently, different concentrations of the test compounds were added and incubated for 24 h. After lysing the cells, they were transferred to 96-well plates, and ATP levels were measured using an ATP assay kit (S0026, Beyotime).

### Oxygen consumption rate

The oxygen consumption rate (OCR) was assessed using a Seahorse XFe96 analyzer (Agilent Technologies). NCI-N87 cells were plated in the provided culture plates at a density of 2.0 × 10^4^ cells per well and incubated overnight. Subsequently, the cells were exposed to the test compounds for 1 h. In line with the Seahorse instrument protocol, the medium was replaced with freshly prepared detection medium using a liquid exchange program. Mitochondrial effectors (oligomycin, Carbonyl cyanide-p-trifluoromethoxyphenylhydrazone (FCCP), and a combination of rotenone and antimycin A) were successively introduced after basal respiration measurements, allowing the evaluation of ATP-linked respiration and spare respiratory capacity. As previously described, the basal OCR was derived by subtracting non-mitochondrial respiration from the final measurement taken prior to oligomycin addition. The maximal OCR was determined upon cell exposure to FCCP, which decouples respiration from ATP synthesis. Maximal respiratory capacity was ascertained by deducting the non-mitochondrial respiration values from the FCCP-induced maximal respiration rates.

### Cellular thermal shift assay

NCI-N87 cells were exposed to 10 μM of compound 4 or DMSO for 1 h. Subsequently, the cells were harvested using PBS supplemented with a protease inhibitor and then aliquoted into individual 0.2 mL PCR tubes, with an average of approximately 1 million cells per tube. Using a PCR instrument (Eppendorf), the samples were subjected to a series of temperature shifts for 3 min at each specified temperature. This freeze-thaw cycle, alternating between liquid nitrogen and 37°C, was repeated thrice. After these cycles, the samples were centrifuged and the supernatant was isolated for western blot analysis.

### Immunoblot analysis

NCI-N87 cells were lysed using radioimmunoprecipitation assay buffer enriched with 1 mM phenylmethylsulfonyl fluoride (PMSF, P0100, Solarbio, China), proteinase inhibitor cocktail, and phosphatase inhibitor cocktail (both from Sigma). Proteins from the lysates were separated using sodium dodecyl sulfate-polyacrylamide gel electrophoresis (SDS-PAGE). Blots were then incubated with specific primary antibodies overnight at 4°C. Membranes were then incubated with secondary antibodies at room temperature for an hour. Finally, proteins were detected on the nitrocellulose membrane using an LI-COR Odyssey infrared imaging system (LI-COR Biotechnology, Lincoln, NE). Phospho-STAT3 (Tyr705) rabbit monoclonal antibodies (AF1276, Beyotime), Stat3 Rabbit Monoclonal antibodies (AF1492, Beyotime), and GAPDH rabbit monoclonal antibodies (AF1186, Beyotime) were used for the analysis.

### Statistical analysis

Experiments were performed in triplicates or more. Student’s t-test was used for statistical evaluation. The significance threshold was set at *P*< 0.05. One-way ANOVA was used to discern the differences between the control and experimental groups. Data are presented as means with 95% confidence intervals, and statistical significance was set at *P*<0.05. All statistical analyses were performed using Microsoft Excel 2016 and GraphPad Prism version 9.

## Results

### Performance evaluation of deep learning models on virtual screening

To identify potential small molecules, we utilized the ComboNet approach for drug-target interactions (DTI) to predict the binding affinity of drugs to STAT3. DTI training data was sourced from the ChEMBL database, which encompasses K biological targets pertinent to the indication or pathogen under investigation. Each DTI dataset contained a roster of molecules paired with their binary DTI labels (positive/negative = 1/0), indicating high and low binding affinities. The affinities are represented as IC_50_ (nM) values. Prior to virtual screening, we modeled the DTI network (as seen in **SI Figure S1**) based on a directional message-passing neural network, consistent with previous studies (22). Specifically for the SH2 domain of STAT3 (illustrated in **SI Figure S2**), our deep learning predictions for STAT3 inhibitors displayed high fidelity (details in **SI Table S1**). These outcomes highlight the optimal performance of the novel STAT3 screening task.

### Virtual screening of STAT3 inhibitors

**Figure 2:**
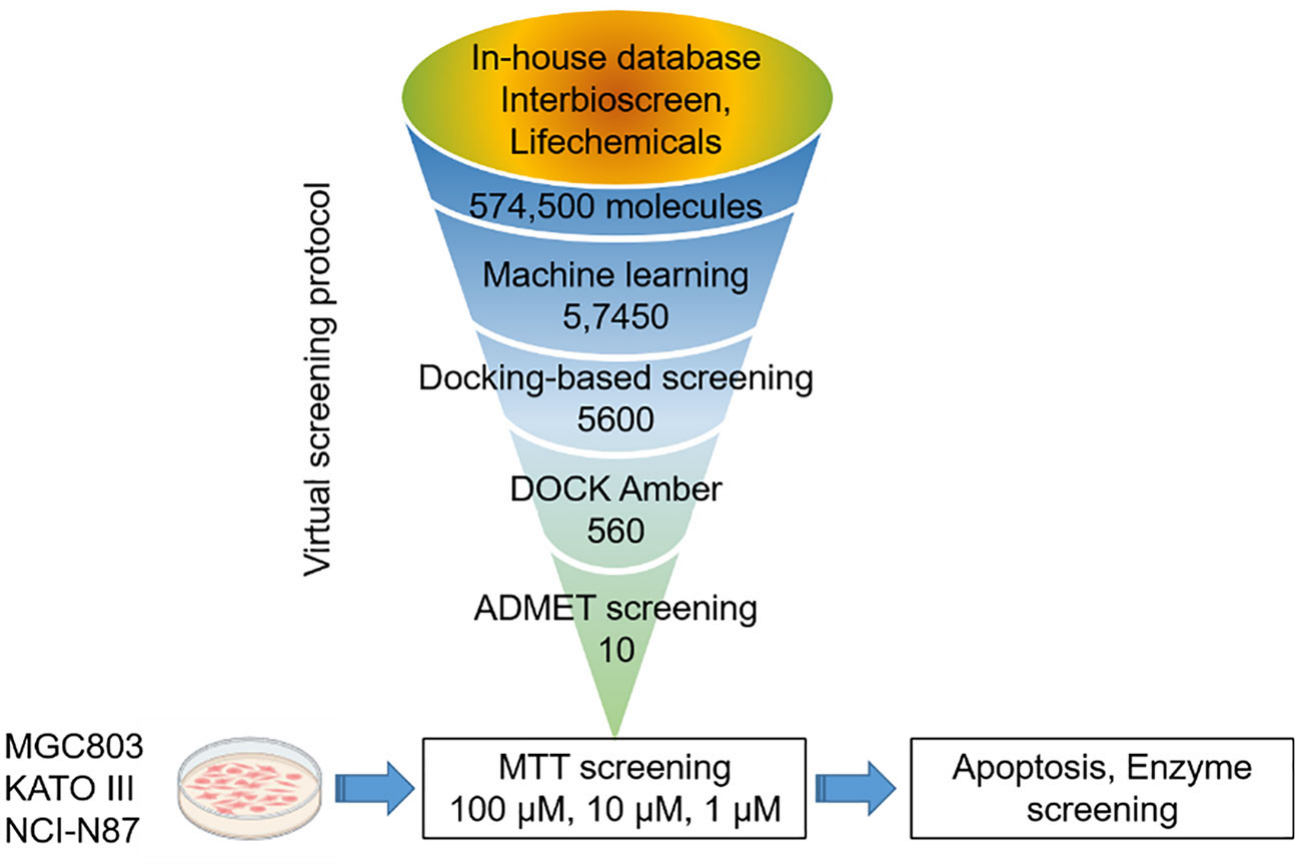
The protocol for virtual screening and biological evaluation of STAT3 inhibitors.

The virtual-screening workflow used in this study is shown in **Figure 2**. By leveraging the robust parallel capabilities of modern CPUs, our deep learning-based DTI model significantly outperforms traditional virtual screening methods that rely on docking scoring. Prior studies have successfully applied deep learning approaches to the virtual screening of expansive compound libraries (25). To expedite our screening process while preserving accuracy, we designed a workflow that integrates deep learning models and conventional docking score functions. This approach facilitates rapid and efficient virtual screening, ensuring a high hit rate across large compound libraries.

**Figure 3:**
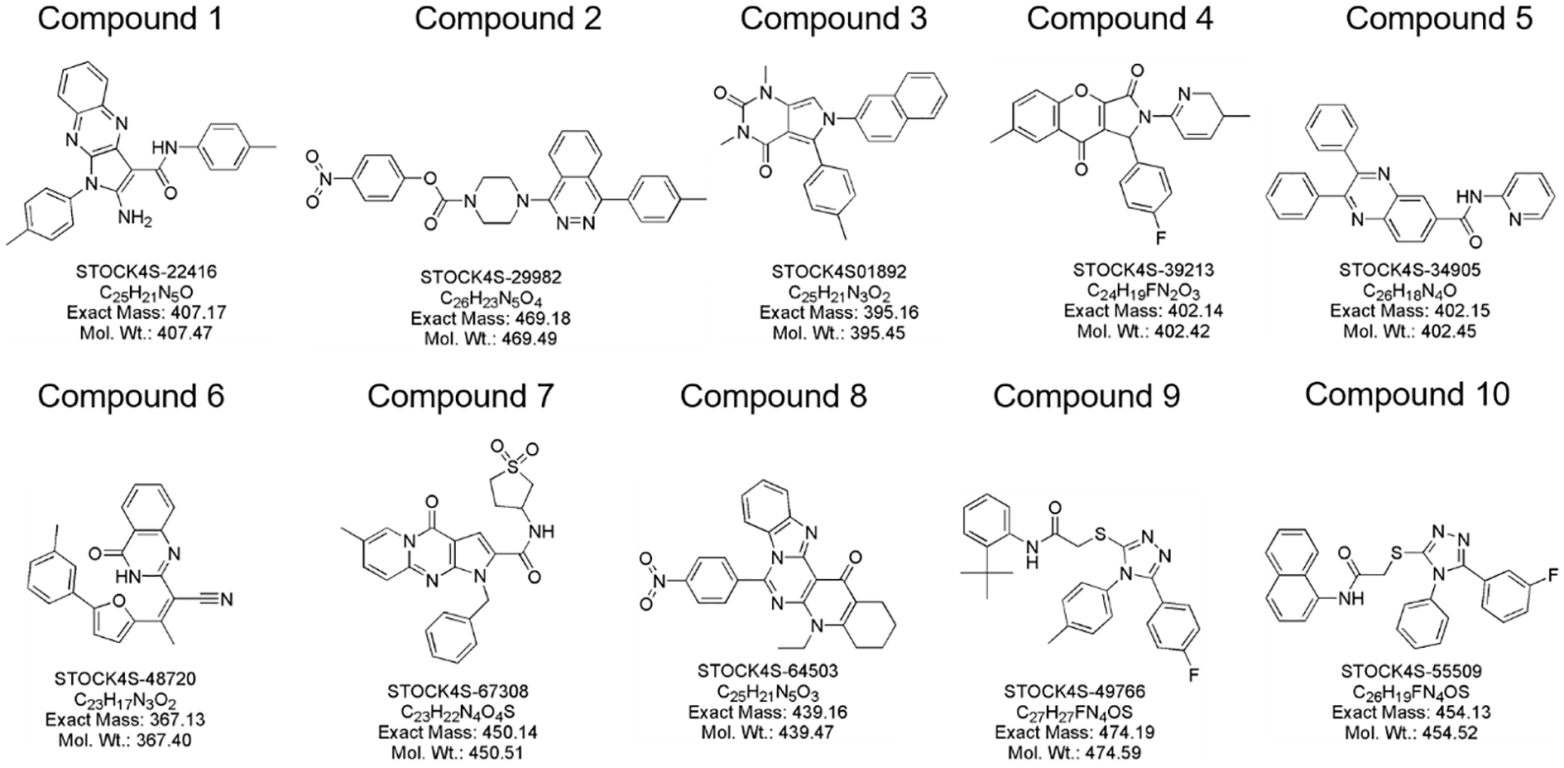
Molecular structure of identified small molecules as potential STAT3 inhibitors based on virtual screening.

In this study, a comprehensive virtual screening endeavor was initiated by curating an extensive library from three distinct molecular databases: our in-house repository, Interioscreen, and Lifechemicals. This holistic compilation comprised an impressive total of approximately 574,500 molecules. Such an extensive selection not only offers a plethora of potential candidates, but also paves the way for a scalable and commercial strategy for subsequent biological activity screening. The target of our docking simulations was STAT3, a pivotal molecule in numerous cellular pathways. The high-resolution structure of STAT3 was obtained from the RSC PDB database (**Supplementary SI Figure S2**). To ensure optimal docking performance, this structure was meticulously refined using the UCSF Chimera software. This entailed the elimination of redundant entities, including specific ions, water molecules, and other non-ligand molecules, which might compromise the docking accuracy. A two-tiered approach was employed to select the most promising STAT3 inhibitors. Initially, a state-of-the-art deep learning algorithm was employed to recognize potential STAT3 inhibitors based on their intricate structural features. This preliminary screening filtered out the top 10% molecules based on their predicted binding affinities. Building on this foundation, structure-based molecular docking was used to further refine selection. Using the sophisticated algorithms of AutoDock Vina, the molecules were ranked based on their binding energy. This subset was further refined by employing the Molecular Mechanics Generalized Born Surface Area (MM-GBSA) method using UCSF DOCK6.10 software, from which the top 10% of the molecules were selected. Consequently, a concise list of ten elite molecules (**Figure 3**) emerged from this rigorous computational gauntlet. These candidates were subsequently subjected to ADMET screening (SI Appendix swissadme_predict files), a crucial step in predicting their pharmacological and toxicological profiles, ensuring that the final molecules chosen were not only potent STAT3 inhibitors but also exhibited favorable drug-like properties.

### *In vitro* cytotoxicity assessment

**Figure 4:**
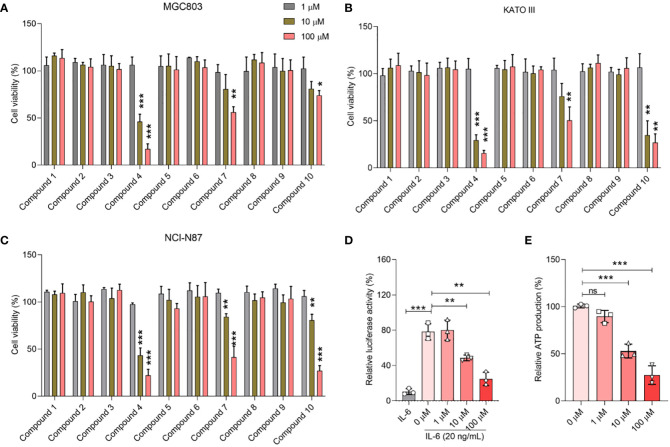
Biological activity of selected compounds on cellular proliferation of MGC803 **(A)**, KATO III **(B)**, and NCI-N87 **(C)**. **(D)** Concentration-dependent inhibition effect of 2a in the STAT3 luciferase assay. **(E)** Concentration-dependent inhibition effect of 2a in the ATP inhibition assay. Data were presented as mean ± SD. Student’s t-testing; ns, no significance; *P < 0.05, ***P < 0.001.

To elucidate the therapeutic potential of the curated compounds, we used the widely acknowledged MTT assay, the gold standard for determining cell viability, specifically targeting gastric cancer cells. As depicted in **Figures 4A–C**, a clear delineation of cellular viability among the three gastric cancer cell lines, MGC803, KATO III, and NCI-N87, was observed when exposed to varying concentrations of the compounds. Notably, compounds 4, 7, and 10 exhibited discernible dose-dependent inhibitory effects on cell proliferation. Conversely, under the experimental conditions, the remaining compounds appeared to spare these gastric cancer cells, exhibiting no significant cytotoxic effects.

Of paramount interest was compound 4, which stood out because of its high efficacy. Remarkably, at a concentration of just 10 μM, this compound managed to curtail the proliferation of gastric cancer cells to below 50%, underscoring its potency. Based on these compelling data, we pivoted our focus to delve deeper into the inhibitory activities of compound 4, specifically targeting its effects on STAT3 within the cellular environment.

### Inhibitory activities on STAT3 *in vitro*

Consistent with prior literature, STAT3 has been implicated in the orchestration of mitochondrial Oxidative phosphorylation (OXPHOS) function, notably through its phosphorylation at the Tyr705 residue. To gain an in-depth understanding of this pivotal modulatory role, we designed a STAT3 luciferase reporter gene assay as a surrogate measure for p-Tyr705 inhibition (26). To complement this, an ATP inhibition assay was also performed to provide a holistic portrayal of STAT3’s functional dynamics. The empirical findings, as shown in **Figures 4D, E**, were compelling. Compound 4 emerged as a potent modulator, exhibiting a profound inhibitory effect on both STAT3 luciferase expression and ATP synthesis, particularly pronounced at a concentration of 10 μM. What captured our attention was the distinct dose-dependent trajectory of compound 4, which systematically attenuated STAT3 luciferase activity along with a concurrent reduction in ATP generation.

### Protein-ligand interaction analysis

**Figure 5:**
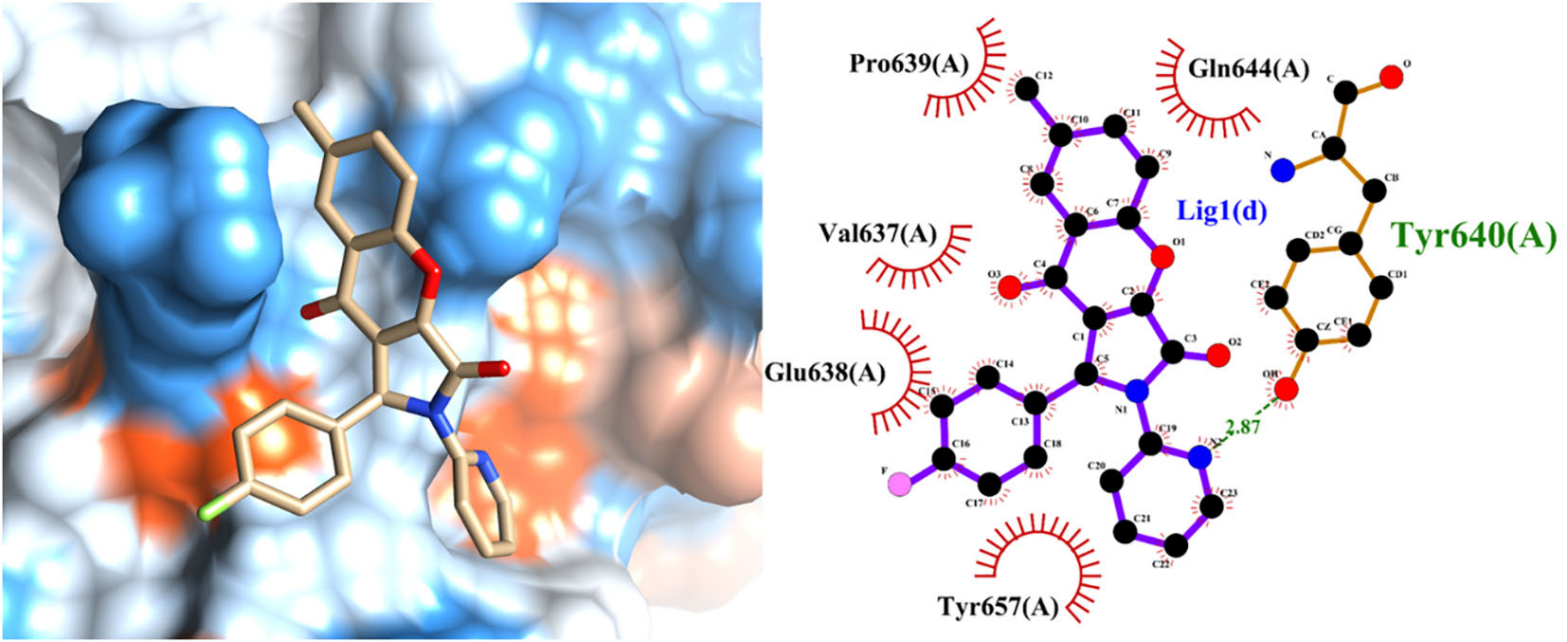
Predicted binding mode of the leading active compound 4 with the STAT3 protein. (Left panel) Surface depiction of the STAT3 binding pocket with the associated ligand; (right panel) Two-dimensional representation of ligand-STAT3 interactions.

To unravel the precise molecular interactions of the previously screened set of ten molecules with STAT3, an intricate molecular docking approach was employed using the sophisticated Autodock4.2 suite. The detailed spatial configurations and presumptive binding modes of these compounds (compounds 1–10) are depicted in **Figure 5**, while the binding poses of the molecular stalwarts among these are delineated in the **Supplementary Information** (**Figures S3-11**). Remarkably, docking insights revealed that the majority of these molecular entities displayed an affinity for the nestle snugly within the STAT3 active pockets, specifically in the SH2 domain. Notably, with the exception of compounds 6 and 9 (**SI Figures S7**, **S10**), the other compounds manifested their potential by forging hydrogen bonds with STAT3. By complementing these, the aromatic moieties of these compounds engage in hydrophobic interactions with the SH2 domain, aligning seamlessly with the active cavities of the domain. In a further delve, compounds 8, 9, and 10 distinguished themselves by creating trio-hydrogen bonds with the STAT3 SH2 domain. These bonds encompassed residues Tyr657, Lys658, and Tyr640 for compounds 8 and 9 and Thr641, Tyr657, and Gln644 for compound 10, providing evidence of their robust interaction potential.

To bolster these findings and ensure their robustness, molecular dynamics simulations of the protein-ligand conjugates were performed using the GROMACS platform. These simulations, depicted in **SI Figure S12**, endorsed the formation of hydrogen bonds, certifying the genuineness and stability of our molecules of interest and the STAT3 protein. Our comprehensive molecular investigations underscored the capability of the screened compounds to forge potent interactions with pivotal residues of STAT3’s SH2 domain. This holds immense promise for their potential as efficacious STAT3 inhibitors.

### Compound 4 inhibits STAT3 phosphorylation at Tyr705

**Figure 6:**
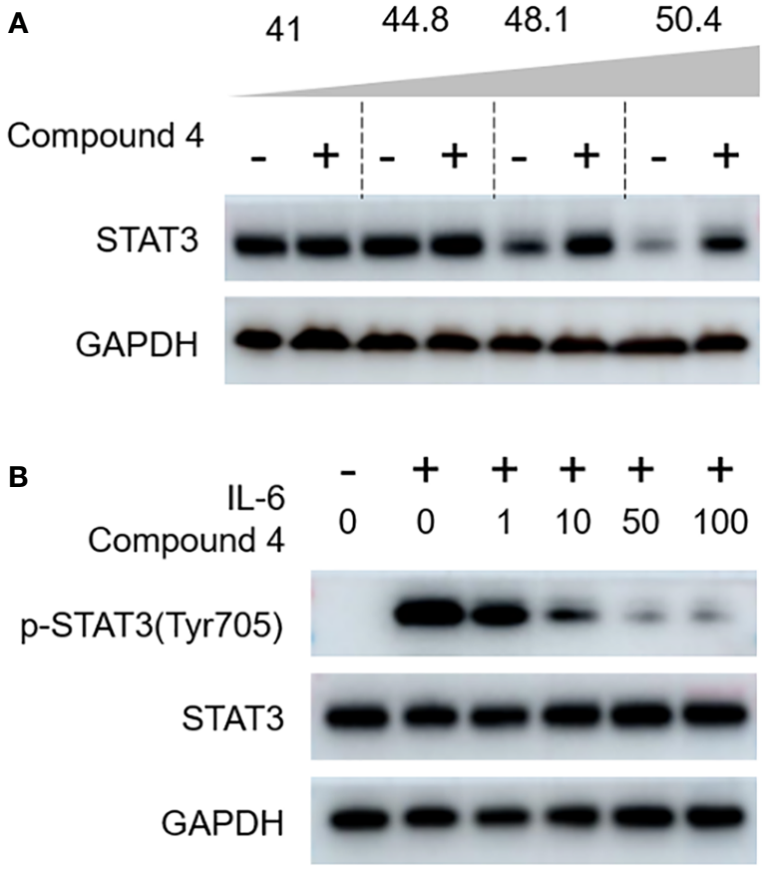
Compound 4 targets STAT3 in gastric cancer cells. **(A)** The interaction of compound 4 with STAT3 was explored by a cellular thermal shift assay in NCI-N87 cells. **(B)** Compound 4 blocks the canonical activation of STAT3 in gastric cancer cells (NCI-N87). Compound 4 abolished IL-6-stimulated STAT3 phosphorylation of Tyr705 in NCI-N87 cells using immunoblot analysis. GAPDH was used as the cytoplasmic control.

Considering the profound attenuation of STAT3 luciferase activity and ATP production, we further investigated to decipher the mechanistic underpinnings of the action of compound 4 on STAT3 via immunoblot analysis. The intricacies of the direct molecular interplay between compound 4 and STAT3 in NCI-N87 cells were revealed using state-of-the-art cellular thermal shift assays (CETSA). **Figure 6A** clearly captures the intriguing observation that STAT3 expression exhibited a consistent decline with increasing incubation temperature when juxtaposed with the DMSO-treated control. However, the narrative shifted dramatically with compound 4 in the mix; a marked stabilization of endogenous STAT3 within NCI-N87 cells emerged, bolstering the notion that compound 4 is closely associated with STAT3 at the cellular level.

Diving deeper, we probed the ramifications of compound 4 on the transcriptional landscape of downstream genes, post-STAT3 inhibition at a 10 μM concentration. Our RT-qPCR analyses, detailed in **Supplementary Information** (**Figure S13**), unequivocally showcased the prowess of compound 4 in thwarting the transcriptional machinery orchestrated by STAT3.

Based on the role of IL-6, a well-known molecular provoker of aberrant STAT3 canonical activation, we sought to delineate the potency of compound 4 in curtailing IL-6-mediated STAT3 phosphorylation at Tyr-705. The immunoblot findings, illustrated in **Figure 6B**, paint a compelling portrait in which IL-6-triggered phosphorylation of Tyr-705 encounters a formidable adversary in compound 4, as evidenced by its dose-dependent inhibitory action.

**Figure 7:**
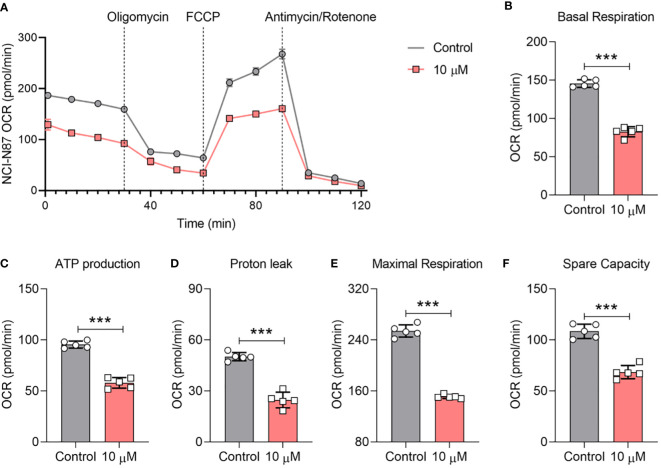
Compound 4 causes mitochondrial OCR depletion in NCI-N87 cells.**(A)** The inhibition effect of the oxygen consumption rate (OCR) was determined by Seahorse after treatment with compound 4. The **(B)** basal OCR, **(C)** ATP production, **(D)** proton leak, **(E)** maximal respiration, and **(F)** spare capacity. Data are presented as Mean ±SD; Student’s t-testing, ***P < 0.001.

Our journey of scientific inquiry further led us to probe compound 4’s influence on the mitochondrial oxidative phosphorylation (OXPHOS) pathway. As prefaced earlier, compound 4 exhibited an uncanny ability to stifle ATP production in gastric cancer cells at a 10 μM concentration. To unpack the broader implications of OXPHOS inhibition within the gastric cancer milieu, we explored the oxygen consumption rate (OCR) in afterexposure to compound 4. **Figure 7** shows that compound 4 emerges as a formidable disruptor of mitochondrial respiration and its inhibitory activity in a dose-dependent manner in NCI-N87 cells. Specific parameters such as basal respiration, ATP linkage, proton leakage, maximal respiration, and spare capacity bore the brunt of the actions of compound 4, exhibiting significant suppression relative to baseline controls (**Figures 7A–F**). In summary, compound 4 effectively disrupted both nuclear transcriptional cascades and mitochondrial OXPHOS pathways, underscoring its potential as a potent inhibitor of STAT3 phosphorylation.

## Discussion

Gastric cancer remains an enigmatic malignancy, notorious for its relentless resistance to existing therapeutic regimens and invariably dismal prognosis (13, 27). This sobering clinical reality underscores the urgent need for the discovery of innovative therapeutic strategies (28). Signal Transducer and Activator of Transcription 3 (STAT3) has emerged through an extensive array of investigations, as a tantalizing therapeutic target for gastric cancer, offering hope in this challenging landscape. However, the design and deployment of effective small-molecule inhibitors targeting STAT3 have proven to be intricate and elusive.

First, we conducted deep learning-integrated virtual screening to identify potential STAT3 inhibitors using molecular docking and ADMET screening methodologies (**Figure 2**). Before virtual screening, a small-molecule library was selected from a commercial database, including our in-house repository, Interioscreen, and Lifechemicals. Interioscreen and Lifechemicals databases have been widely utilized for virtual screening to identify potential kinase inhibitors, including Epidermal Growth Factor Receptor (EGFR) (29), FOXO3a (30), and nicotinic acid phosphoribosyltransferase (NAPRT) (31). Our selection of small-molecule libraries contributed to the identification of potential STAT3 inhibitors for further biological evaluation. Structure-based virtual drug screening has become the cornerstone of drug discovery owing to its cost-effectiveness and high efficiency (32). Consequently, we utilized the ComboNet approach for drug-target interactions (DTI) to reduce the population of compound candidates for potential STAT3 inhibitors. This approach has been successfully employed to identify kinase inhibitors (33, 34). The top 10% of the small molecules were selected for the next molecular docking process. Subsequently, Autodock vina and UCSF DOCK6.0, with MM-GBSA, were further employed to screen the library from ~57,400 to 560 small molecules. ADMET screening was used to select the top 10 small molecules for further biological evaluation. This screening step enriched the abundance of potential STAT3 in the small-molecule library.

To evaluate the biological activities of the selected compounds, three gastric cancer cell lines, *i.e.* MGC803, NCI-N87, and KATO III, were used to determine whether the compounds could suppress the proliferation of gastric cancer cells. Screening by gradient concentration from 1 μM to 100 μM, compounds 4, 7 and 10 could inhibit proliferation of these gastric cancer cells (**Figure 4**), especially for compound 4. Compound 4 is a typical γ-benzopyrone moieties, which were widely used as pharmacophore of anticancer drugs (35, 36), and the ADMET properties of compound 4 also displayed the potential to be as lead compound (SI swissadme_predict.csv). The STAT3 luciferase assay clearly demonstrated that compound 4 bindsto STAT3 at the cellular level (**Figure 4D**). STAT3 expression is strongly associated with mitochondria respiration (37–39). Subsequently, we examined ATP production in gastric cancer cells after treatment with various concentrations of compound 4. These results clearly indicated that compound 4 interfered with mitochondrial function. Therefore, examination of the oxygen consumption rate in cancer cells treated with compound 4 will provide further insight into the biological effects of compound 4 on cellular mitochondrial respiration.

To date, oxygen consumption rate (OCR) measurements have been widely used to examine STAT3 inhibitors at cell levels (40, 41). We also examined the OCR curve of gastric cancer cells after treatment with compound 4 (**Figure 7**), which indicated that compound 4 could affect the mitochondrial respiration of gastric cancer cells by inhibiting STAT3. Analysis of basal respiration, ATP production, proton leakage, maximal respiration, and spare capacity showed that compound 4 strongly suppressed these processes (**Figures 4B–F**). Oligomycin A is an ATPase inhibitor that directly produces ATP. **Figure 7C** shows that the inhibition of STAT3 could affect ATPase activity, which is consistent with previous reports that STAT3 associates with H^+^-ATPase (42). Moreover, several studies have reported that STAT3 affects the electron transport chain to regulate cell proliferation (43), chemosensitivity (44). The suppression of electron transport chain activity (**Figures 7B, E, F**) also implied that compound 4 may be a potential treatment to rescue chemotherapy resistance in the future.

The SH2 domain of STAT3 is not just a mere functional entity but is its most conserved and pivotal domain (45). It orchestrates the intricate choreography of STAT3 dimerization and cognition of upstream kinases. Phosphorylation of STAT3 at Tyr705 ushers in its dimer formation, serving as a prelude to its translocation to the nucleus and triggering a cascade of transcriptional events. Based on our Fluorescence Polarization (FP) assay outcomes, which illuminated the ability of compound 4 to displace the specific STAT3-SH2 binding peptide *in vitro* (as depicted in **Figure 4D**), we postulated a direct affinity of compound 4 to the STAT3-SH2 domain. This hypothesis was confirmed by molecular docking studies. Indeed, these docking endeavors underscore the interactions of compound 4 with a myriad of amino acid residues within STAT3-SH2, notably Val637, Glu638, Pro639, Gln644, Tyr640, and Tyr657. This hypothesis was further supported by the Cellular Thermal Shift Assay (CETSA), reinforcing the allegiance of compound 4 to STAT3-SH2.

## Conclusion

STAT3 is known to play a critical role in the proliferation of gastric cancer cells, which makes it a promising target for the development of antitumor drugs. We successfully identified one novel and efficient STAT3 inhibitor using a structure-based virtual screening strategy. *In vitro* cytotoxicity, immunoblot analysis, and OCR testing clearly revealed that this compound not only had strong inhibitory activities on the STAT3 protein, but also exhibited high selectivity for gastric cancer cells. Structural optimization of compound 4 will be further carried out by our group to develop novel STAT3 inhibitor drugs for clinical treatment.

## Data availability statement

The original contributions presented in the study are included in the article/**Supplementary Material**. Further inquiries can be directed to the corresponding authors.

## Author contributions

WL: Conceptualization, Data curation, Formal analysis, Investigation, Methodology, Project administration, Resources, Software, Validation, Visualization, Writing – original draft. ZC: Conceptualization, Data curation, Investigation, Methodology, Validation, Writing – original draft. CY: Conceptualization, Data curation, Formal analysis, Resources, Writing – review & editing. TY: Data curation, Formal analysis, Investigation, Methodology, Writing – review & editing. YY: Methodology, Resources, Validation, Visualization, Writing – review & editing. HW: Conceptualization, Data curation, Investigation, Methodology, Writing – original draft, Writing – review & editing. JS: Formal analysis, Validation, Visualization, Writing – original draft.
