# Supplementary material for: Discovery of potent STAT3 inhibitors using structure-based virtual screening, molecular dynamic simulation, and biological evaluation
Source: Front Oncol. 2023 Nov 2;13:1287797. doi: 10.3389/fonc.2023.1287797 (PMC10652556; doi:10.3389/fonc.2023.1287797)
Supplement: Supplementary file 1 [file DataSheet_1.pdf]

Supplementary information

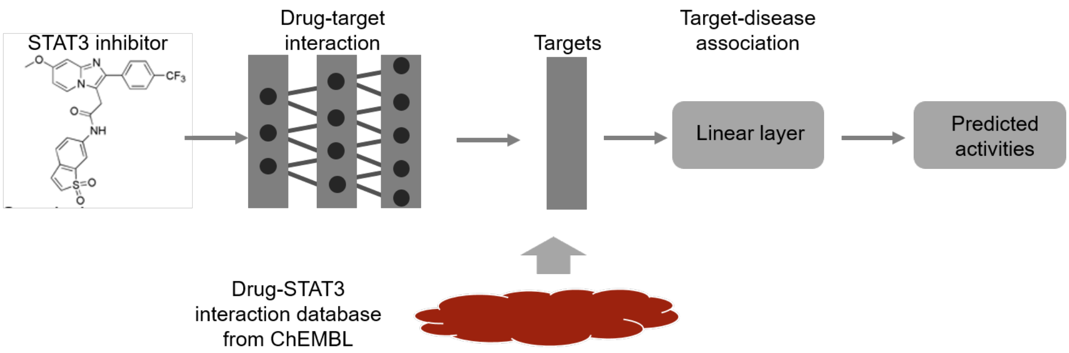

SI Fig.S1. ComboNet for synergistic drug combination discovery. ComboNet is composed of two networks: a DTI and a target−disease association network. The antiviral effect of a single drug is predicted from its representation. The vector characterizes the DTI features of drug A.

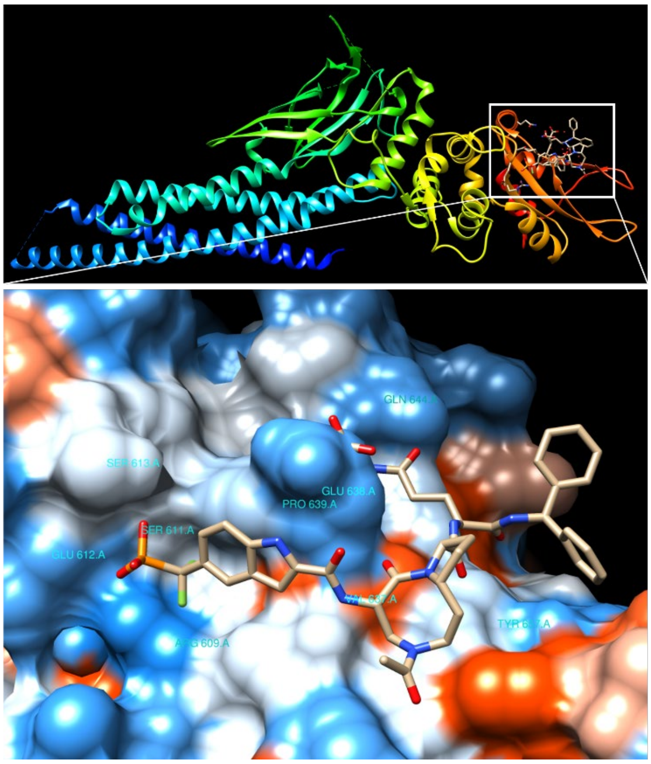

SI Fig.S2. Structure of STAT3 bound to small molecular inhibitor (PBD ID 6NJS). SH2 domain of STAT3 represent the hydrophilic areas.

SI Table S1. Validation, test and screening set results.

	Specificity	Sensitivity	ROC-AUC
Valid	0.836	0.802	0.901
Test (all)	0.861	0.833	0.922

Test (Hard)	0.858	0.827	0.912
Screen	0.682	1.00	0.856

SI Table S2. SMILES format of selected compound in this study.

Name	SMILES
Compound 1	c1c(ccc(c1)n1c(c(c2c1nc1c(n2)cccc1)C(=O)Nc1ccc(cc1)C)N)C
Compound 2	c1c(ccc(c1)C)c1c2c(c(nn1)N1CCN(CC1)C(=O)Oc1ccc(cc1)N(=O)=O)cccc2
Compound 3	c1(=O)n(c(=O)c2c(n1C)cn(c2c1ccc(cc1)C)c1ccc2c(c1)cccc2)C
Compound 4	c1c(cc2c(c1)oc1c(c2=O)C(N(C1=O)C1=NCC(C=C1)C)c1ccc(cc1)F)C
Compound 5	c1c(cc2c(c1)nc(c(n2)c1ccccc1)c1ccccc1)C(=O)Nc1ncccc1
Compound 6	c1ccc(cc1C)c1ccc(o1)/C(=C(/C#N)\c1nc2c(c(=O)[nH]1)cccc2)/C
Compound 7	c1(ccc2n(c1)c(=C)c1c(n2)n(c(c1)C(=O)NC1CS(=O)(=O)CC1)Cc1ccccc1)C
Compound 8	c1ccc2c(c1)nc1n2c(nc2c1c(=O)c1c(n2CC)CCCC1)c1ccc(cc1)N(=O)=O
Compound 9	c1cc(c(cc1)NC(=O)CSc1n(c(nn1)c1ccc(cc1)F)c1ccc(cc1)C)C(C)(C)C
Compound 10	c1ccc2c(c1)c(ccc2)NC(=O)CSc1n(c(nn1)c1cccc(c1)F)c1ccccc1

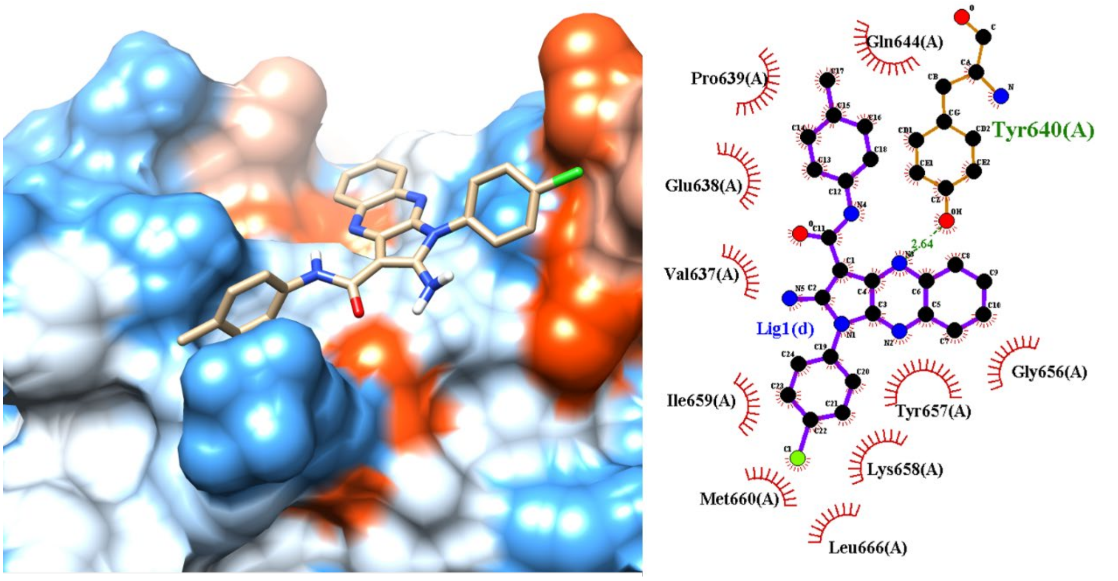

SI Fig. S3. Predicted binding mode of the leading active compound 1 with the STAT3 protein. (Left panel) Surface depiction of the STAT3 binding pocket with the associated ligand; (right panel) Two-dimensional representation of ligand-STAT3 interactions.

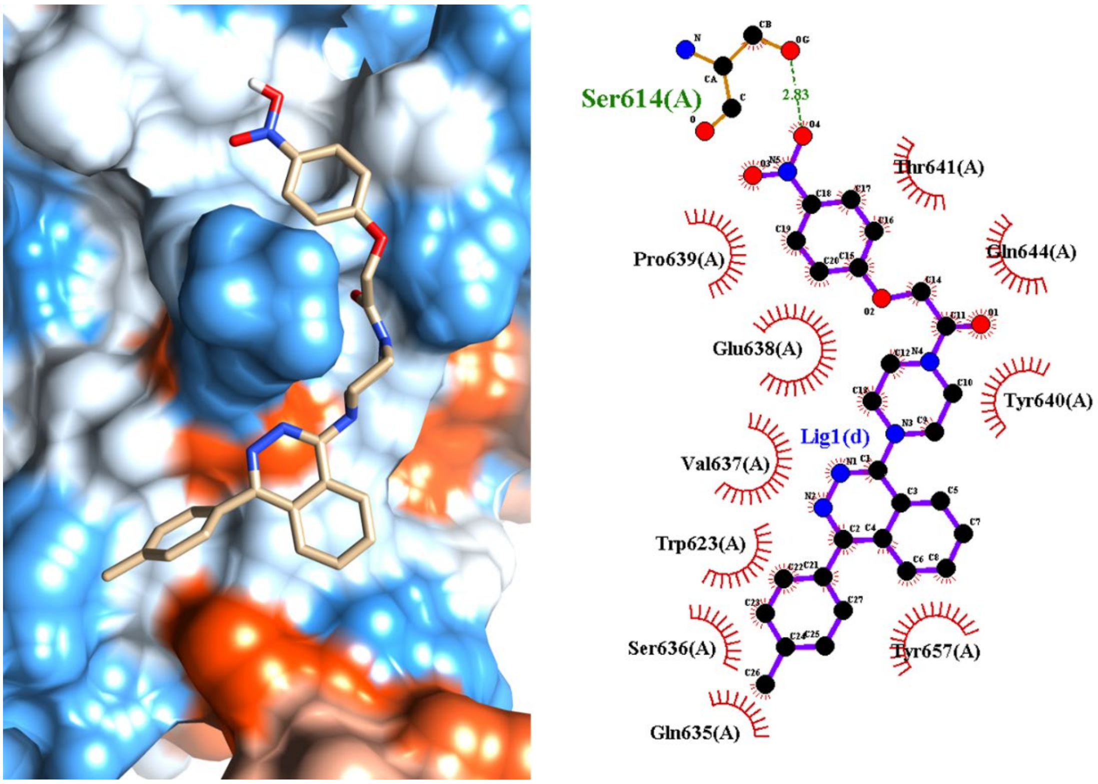

SI Fig. S4. Predicted binding mode of the leading active compound 2 with the STAT3 protein. (Left panel) Surface depiction of the STAT3 binding pocket with the associated ligand; (right panel) Two-dimensional representation of ligand-STAT3 interactions.

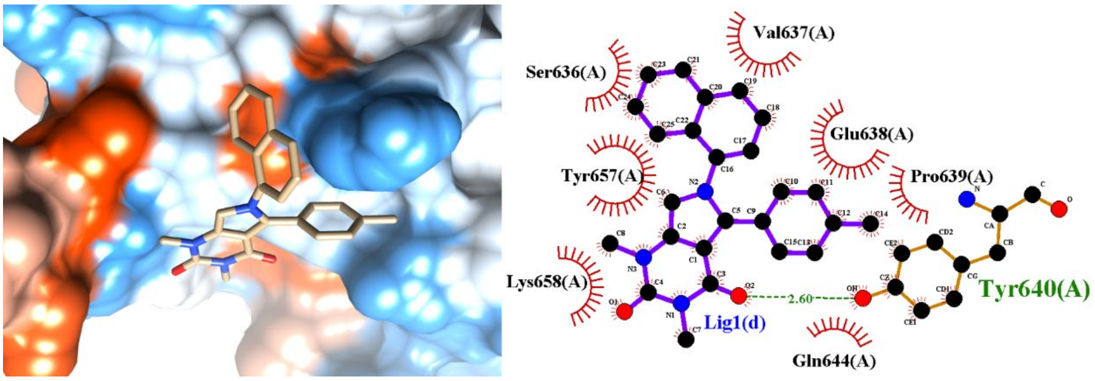

SI Fig. S5. Predicted binding mode of the leading active compound 3 with the STAT3 protein. (Left panel) Surface depiction of the STAT3 binding pocket with the associated ligand; (right panel) Two-dimensional representation of ligand-STAT3 interactions.

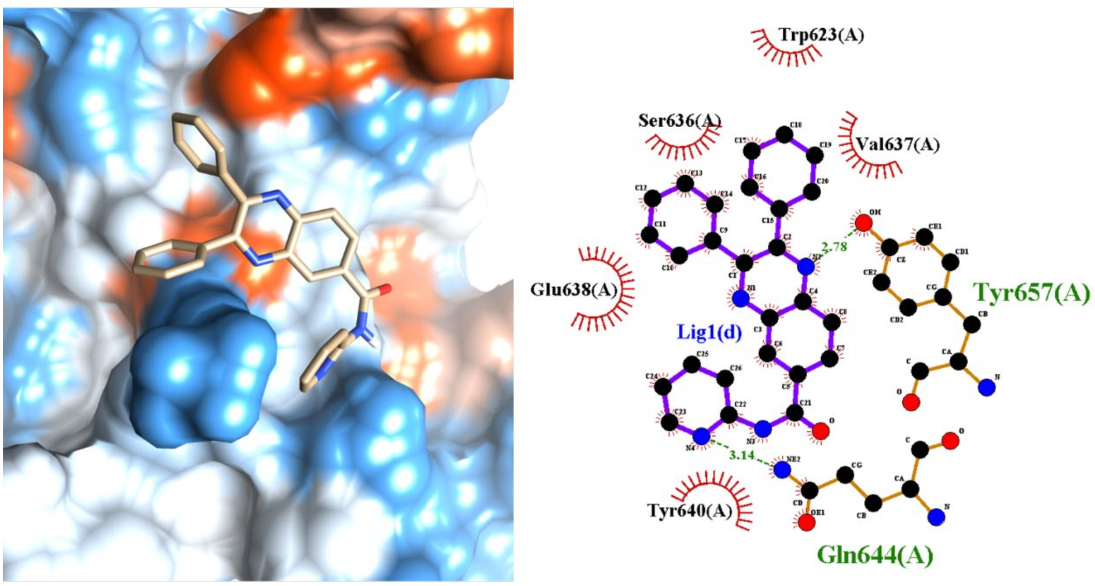

SI Fig. S6. Predicted binding mode of the leading active compound 5 with the STAT3 protein. (Left panel) Surface depiction of the STAT3 binding pocket with the associated ligand; (right panel) Two-dimensional representation of ligand-STAT3 interactions.

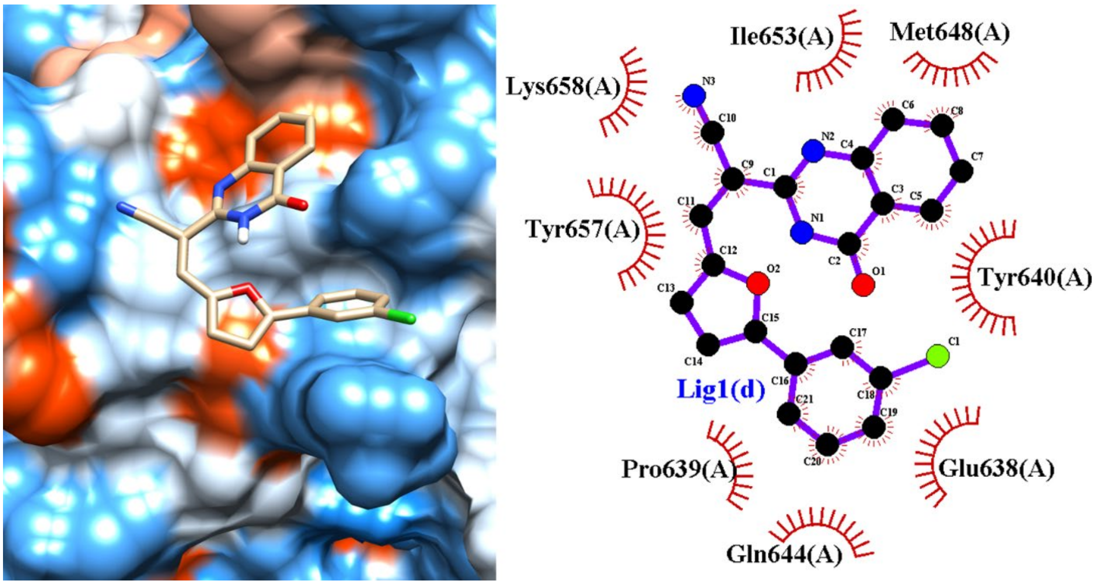

SI Fig. S7. Predicted binding mode of the leading active compound 6 with the STAT3 protein. (Left panel) Surface depiction of the STAT3 binding pocket with the associated ligand; (right panel) Two-dimensional representation of ligand-STAT3 interactions.

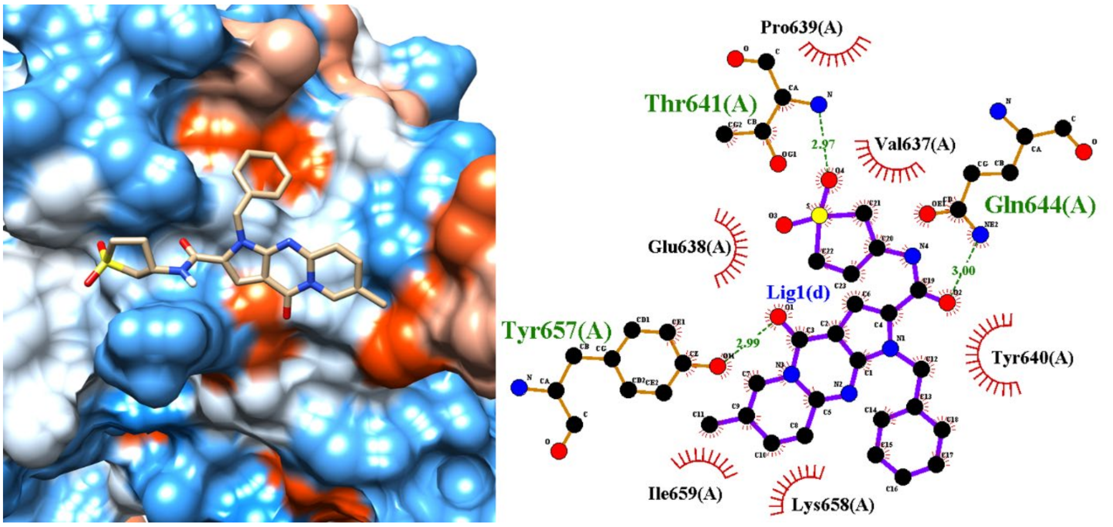

SI Fig. S8. Predicted binding mode of the leading active compound 7 with the STAT3 protein. (Left panel) Surface depiction of the STAT3 binding pocket with the associated ligand; (right panel) Two-dimensional representation of ligand-STAT3 interactions.

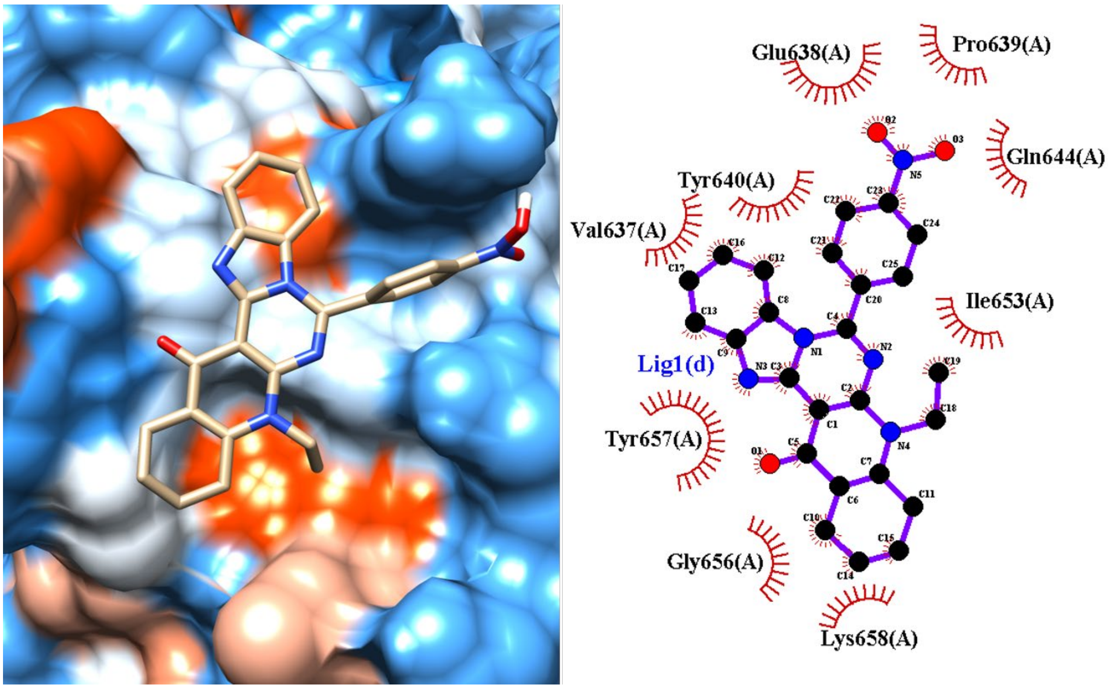

SI Fig. S9. Predicted binding mode of the leading active compound 8 with the STAT3 protein. (Left panel) Surface depiction of the STAT3 binding pocket with the associated ligand; (right panel) Two-dimensional representation of ligand-STAT3 interactions.

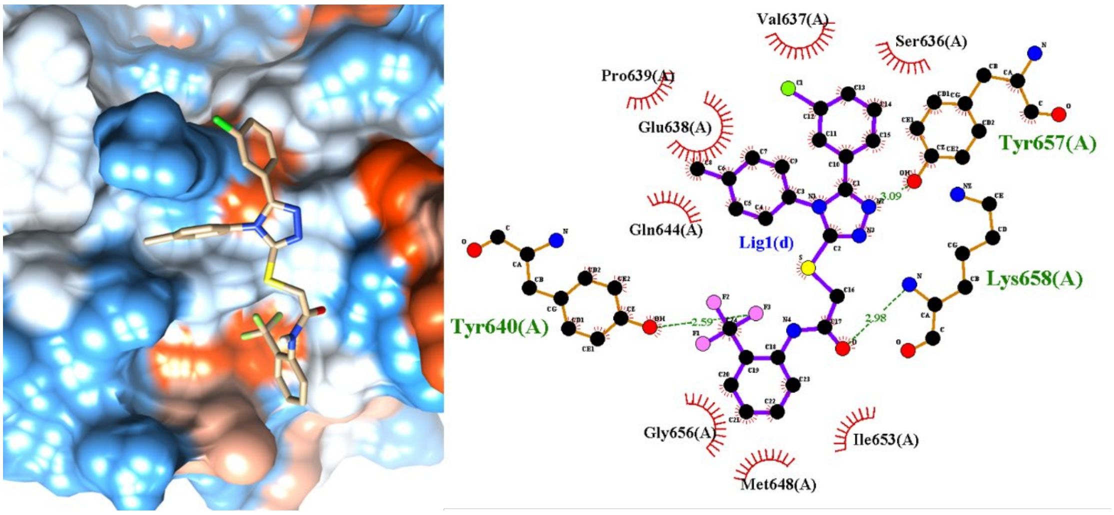

SI Fig. S10. Predicted binding mode of the leading active compound 9 with the STAT3 protein. (Left panel) Surface depiction of the STAT3 binding pocket with the associated ligand; (right panel) Two-dimensional representation of ligand-STAT3 interactions.

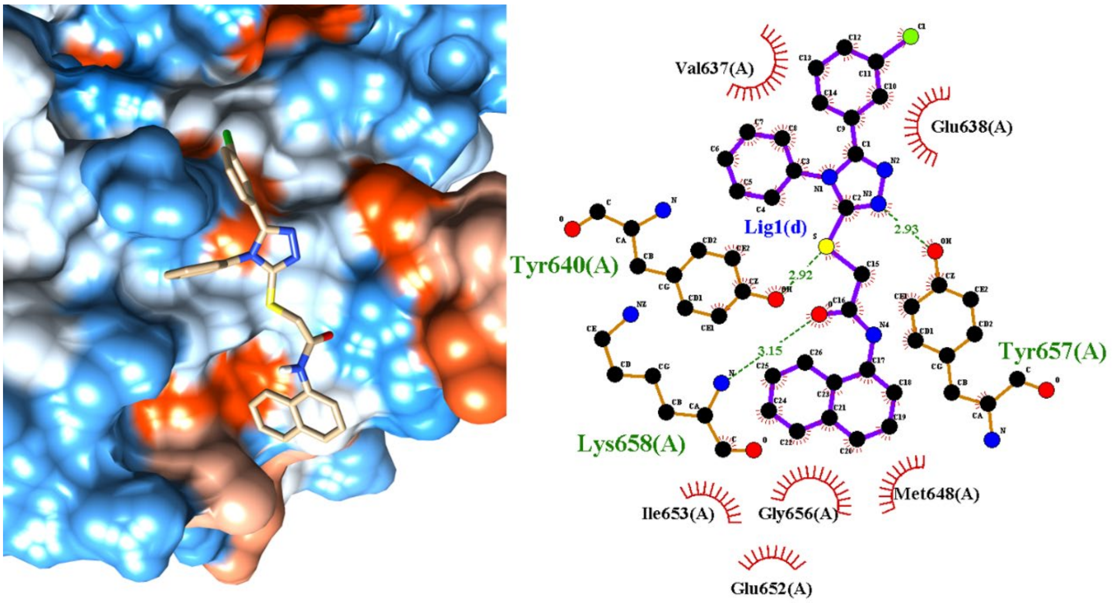

SI Fig. S11. Predicted binding mode of the leading active compound 10 with the STAT3 protein. (Left panel) Surface depiction of the STAT3 binding pocket with the associated ligand; (right panel) Two-dimensional representation of ligand-STAT3 interactions.

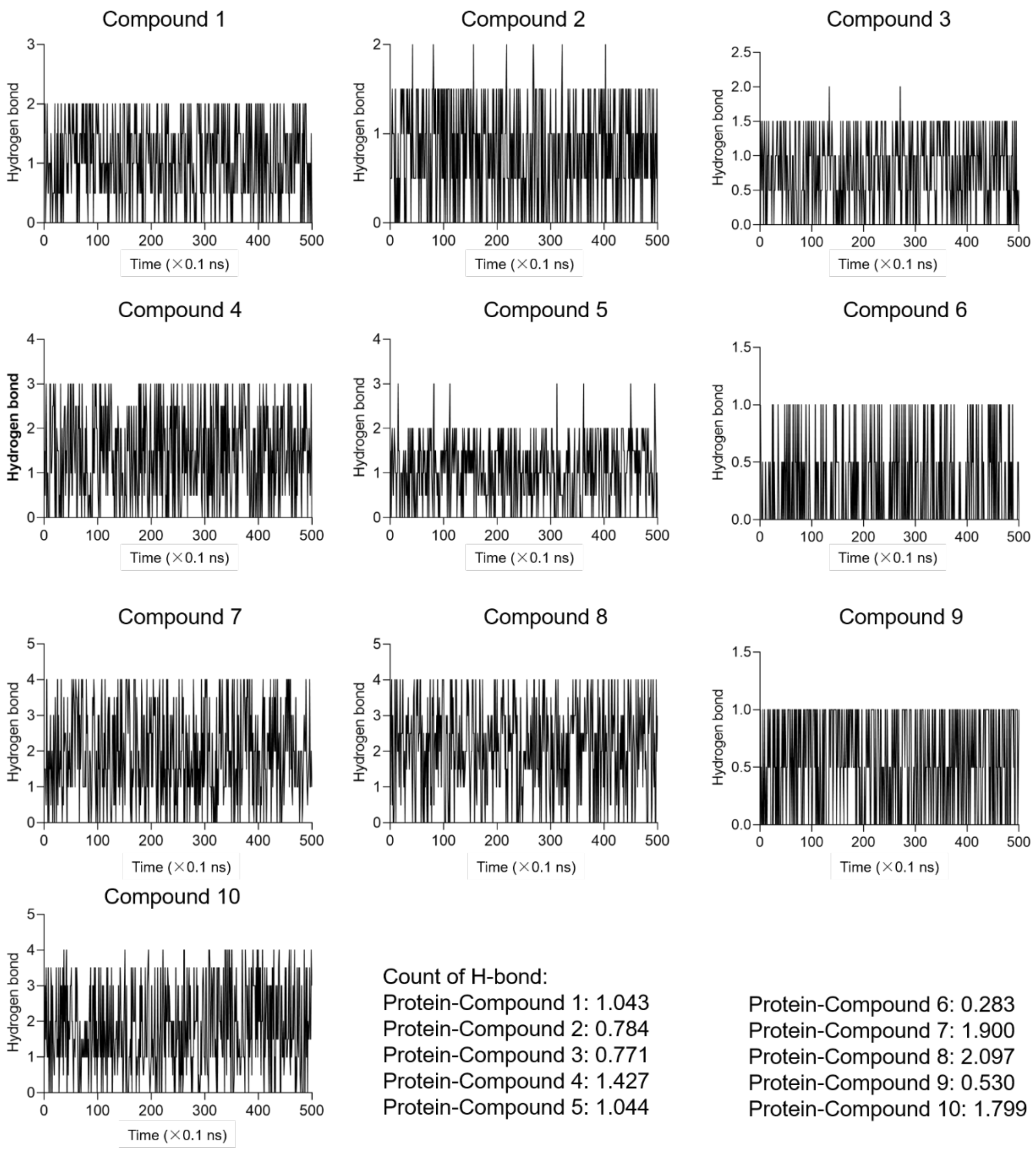

Si Fig.S12. H-bond interaction analysis of STAT3 and selected inhibitors with crucial residues of SH2 domain during molecular dynamic simulation in 50 ns.

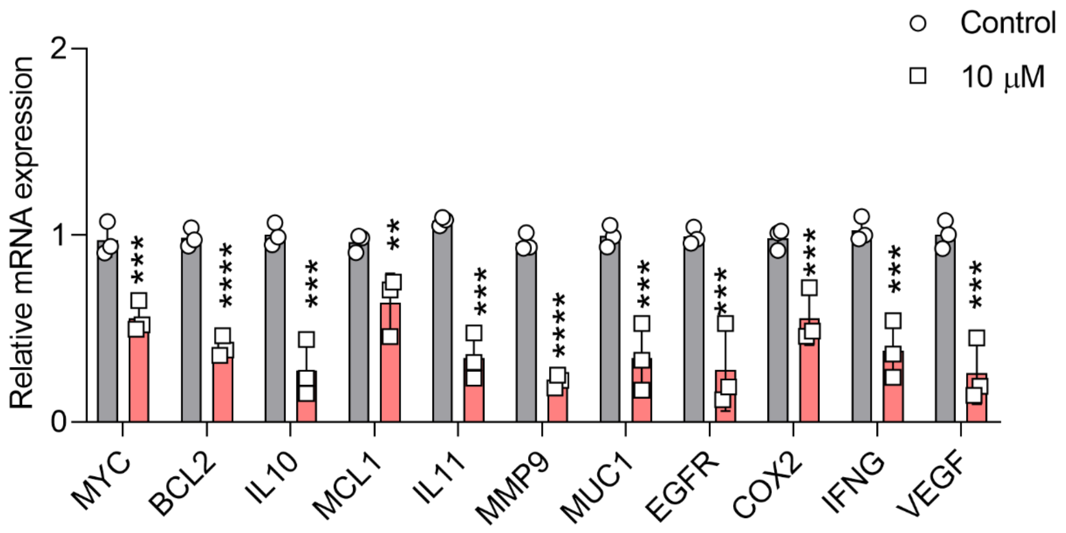

SI Fig.S13. Quantification expression levels of down-stream genes modulated by STAT3 factor in NCI-N87 for 24 h. Data are presented as Mean ± SD. Student's t-testing, *** $P < 0.001$, **** $P < 0.0001$.

Experimental procedure:

To ascertain the mRNA levels, both semi-quantitative and real-time reverse transcription-polymerase chain reaction (RT-PCR) were employed, drawing on established methodologies. The procedure commenced with the extraction of total RNA from cellular samples employing the TRIzol reagent (Invitrogen), strictly adhering to the guidelines provided by the manufacturer. Subsequently, 1 μg of this isolated RNA served as the blueprint for cDNA synthesis, facilitated by the M-MLV reverse transcriptase (sourced from Promega). The real-time RT-PCR analyses were meticulously executed on a state-of-the-art LightCycler 480II detection system (a product of Roche) while employing the LightCycler 480 SYBR Green I Master Mix (also from Roche). To ensure accuracy and context, the expression metrics of the target genes were harmonized against the consistent expression of the housekeeping gene, GAPDH.

SI Table S3. Primer sequence of STAT3-targeted genes.

Gene	Forward	Reverse
MYC	CCTGGTGCTCCATGAGGAGAC	CAGACTCTGACCTTTTGCCAGG
VEGF	GTACCTCCACCATGCCAAGT	AATAGCTGCGCTGGTAGACG
BCL2	ATCGCCCTGTGGATGACTGAGT	GCCAGGAGAAATCAAACAGAGGC
IL10	TCTCCGAGATGCCTTCAGCAGA	TCAGACAAGGCTTGGCAACCCA
MCL1	CCAAGAAAGCTGCATCGAACCAT	CAGCACATTCCTGATGCCACCT
IL11	GGACCACAACCTGGATTCCCTG	AGTAGGTCCGCTCGCAGCCTT
MMP9	GCCACTACTGTGCCTTTGAGTC	CCCTCAGAGAATCGCCAGTACT
MUC1	CCTACCATCCTATGAGCGAGTAC	GCTGGGTTTGTGTAAGAGAGGC
EGFR	AACACCCTGGTCTGGAAGTACG	TCGTTGGACAGCCTTCAAGACC
COX2	CGGTGAAACTCTGGCTAGACAG	GCAAACCGTAGATGCTCAGGGA
GAPDH	GTCTCCTCTGACTTCAACAGCG	ACCACCCTGTTGCTGTAGCCAA
